# Supplementary material for: Defining drivers of under-immunization and vaccine hesitancy in refugee and migrant populations
Source: J Travel Med. 2023 Jun 19;30(5):taad084. doi: 10.1093/jtm/taad084 (PMC10481413; doi:10.1093/jtm/taad084)
Supplement: Supplementary_material_1_taad084 [file supplementary_material_1_taad084.docx]

**Table 1.** Search Strategy

| **Theme** | **Keywords** |
| --- | --- |
| Migrants | migrant* OR immigrant* OR emigrant* OR foreign* OR asylum* OR asylee* OR refugee* non-citizen* OR citizenship OR nationality OR undocumented OR non-resident* OR expat* OR newcomer* |
| Vaccination | vaccin* or immunis* or immuniz* |
| Drivers of under-immunisation and vaccine hesitancy | (uptake or demand or coverage or utiliz* or utilis*) OR  (barrier* or enabl* or facilitat* or motivat* or obstacle* or determinant* or factor* or reason* or challenge*) OR  (accept* or comply or complian* or adher* or readiness or intent* or willing*) OR  (avoid* or refus* or hesita* or renounc* or reject* or deny or deni* or delay*) OR  (confiden* or trust* or fear* or wary or wariness or doubt* or sceptic* or concern* or complacen*) OR  (attitude* or perception* or perspective* or view* or belief*) OR  (practice* or behaviour*) OR (decision or decision-making) OR  (aware* or knowledge* or inform* or understand*) OR  (access* or cost* or afford* or navigat* or availab*) |
| Years | 2010 – 2022 |
